# Ionic Liquid-In-Oil Microemulsions Prepared with Biocompatible Choline Carboxylic Acids for Improving the Transdermal Delivery of a Sparingly Soluble Drug

**DOI:** 10.3390/pharmaceutics12040392

**Published:** 2020-04-24

**Authors:** Md. Rafiqul Islam, Md. Raihan Chowdhury, Rie Wakabayashi, Noriho Kamiya, Muhammad Moniruzzaman, Masahiro Goto

**Affiliations:** 1 Department of Applied Chemistry, Graduate School of Engineering, Kyushu University, 744 Motooka, Nishi-ku, Fukuoka 819-0395, Japan; islam.md.334@s.kyushu-u.ac.jp (M.R.I.); chowdhury.md.raihan.588@m.kyushu-u.ac.jp (M.R.C.); rie_wakaba@mail.cstm.kyushu-u.ac.jp (R.W.); kamiya.noriho.367@m.kyushu-u.ac.jp (N.K.); 2 Department of Applied Chemistry and Chemical Engineering, Noakhali Science and Technology University, Noakhali 3814, Bangladesh; 3 Advanced Transdermal Drug Delivery System Center, Kyushu University, 744 Motooka, Nishi-ku, Fukuoka 819-0395, Japan; 4 Division of Biotechnology, Center for Future Chemistry, Kyushu University, 744 Motooka, Nishi-ku, Fukuoka 819-0395, Japan; 5 Chemical Engineering Department, Universiti Teknologi PETRONAS, Seri Iskandar 32610, Perak, Malaysia; m.moniruzzaman@utp.edu.my

**Keywords:** biocompatible, ionic liquid, transdermal drug delivery system, microemulsion

## Abstract

The transdermal delivery of sparingly soluble drugs is challenging due to of the need for a drug carrier. In the past few decades, ionic liquid (IL)-in-oil microemulsions (IL/O MEs) have been developed as potential carriers. By focusing on biocompatibility, we report on an IL/O ME that is designed to enhance the solubility and transdermal delivery of the sparingly soluble drug, acyclovir. The prepared MEs were composed of a hydrophilic IL (choline formate, choline lactate, or choline propionate) as the non-aqueous polar phase and a surface-active IL (choline oleate) as the surfactant in combination with sorbitan laurate in a continuous oil phase. The selected ILs were all biologically active ions. Optimized pseudo ternary phase diagrams indicated the MEs formed thermodynamically stable, spherically shaped, and nano-sized (<100 nm) droplets. An in vitro drug permeation study, using pig skin, showed the significantly enhanced permeation of acyclovir using the ME. A Fourier transform infrared spectroscopy study showed a reduction of the skin barrier function with the ME. Finally, a skin irritation study showed a high cell survival rate (>90%) with the ME compared with Dulbecco’s phosphate-buffered saline, indicates the biocompatibility of the ME. Therefore, we conclude that IL/O ME may be a promising nano-carrier for the transdermal delivery of sparingly soluble drugs.

## 1. Introduction

The transdermal drug delivery system (TDDS), a safe, non-invasive, easy, and effective drug delivery system, has attracted much attention in recent research because of its numerous prospective advantages, including improved patient compliance, avoidance of the first-pass metabolism, persistent and controlled delivery, and reduction of undesirable adverse effects [1,2]. However, the widespread use of this system is restricted to a few drugs only, because of the impermeable nature of the stratum corneum (SC), the outermost layer of the skin [3,4]. To overcome these limitations, many micro-structured fluid systems, including microemulsions, nanoparticles, and permeation enhancers containing other vesicles have been investigated to improve drug delivery efficiency via disrupting and modifying the regular arrangement of the corneocytes of the SC [5,6]. Among these fluid systems, attention has been focused on microemulsions (MEs), usually consisting of water, oil, and surfactant, which are useful colloidal nano-carriers for a TDDS, owing to their thermodynamic stability, high drug-loading capacity, and very low surface tension [7,8]. However, the conventional water-in-oil (W/O) and oil-in-water (O/W) ME systems are not suitable for drugs that are insoluble or sparingly soluble in water and most organic solvents [8,9,10].

Ionic liquids (ILs) are organic salts consisting of organic cations and inorganic and/or organic anions that have melting points below 100 °C. Because of their various unique physicochemical properties, ILs have become important in diverse scientific and technological arenas, especially used in ME systems in all the phases, formed by altering the water, oil, and surfactants [8,9,11]. The first IL-in-oil (IL/O) ME was developed by Moniruzzaman et al., in which the core aqueous phase was replaced by a hydrophilic IL, dimethylimidazolium dimethylphosphate ([C1mim][DMP]), which has emerged as a nano-carrier with great potential in the field of TDDSs for its excellent solubilizing capacity of sparingly soluble drugs [8,9]. Later other researchers have also used imidazolium ILs as the polar phase to increase the solubility and permeability of drugs [11,12]. However, most of the IL/O MEs were prepared with polyoxyethylene sorbitan monooleate (Tween-80), sorbitan laurate (Span-20), and other conventional non-ionic surfactants that require a high amount of surfactant for drug loading, which reduces the permeability [13], and increases the toxicity of the MEs [14]. Recently, surface-active ILs (SAILs) have been introduced as ILs, which act as surfactants to increase the physico-thermal stability [15,16] and the permeability of MEs [11].

Despite having various advantages, the use of ILs is limited because of their high toxicity and low biocompatibility and degradability [17]. In fact, most of the ILs used in previous studies, including imidazolium, pyridinium, and quinolinium cations and high strength inorganic acid anion-based ILs cannot be used in clinical applications because of their high toxicity and low biocompatibility [18,19]. Interestingly, ILs containing choline and amino acid esters as cations and organic acid anions (e.g., acetate, phosphate, and carboxylate) are non-toxic, biocompatible, and biodegradable [10,18,20]. It has been reported that ILs containing choline as the cation were less toxic compared with ILs containing imidazolium cations [21,22]. ILs containing choline as the cation and carboxylic acid as the anion are generally regarded as safe (GRAS), and considered to be non-toxic and biodegradable because of their biological sources [23,24,25].

In this study, we prepared IL/O MEs, using biocompatible ILs as replacements for imidazolium-based ILs, for the transdermal delivery of acyclovir (ACV), a model sparingly soluble antiviral drug. We selected choline formate ([Ch][For]), choline lactate ([Ch][Lac]), and choline propionate ([Ch][Pro]) ILs as the non-aqueous polar phases in the core of the MEs. In addition, a long chain (C_18_) fatty acid SAIL, choline oleate ([Ch][Ole]) was incorporated as the surfactant in combination with Span-20 in a continuous oil phase of isopropyl myristate (IPM). The ILs and SAIL were selected because of their biocompatibility and negligible toxicity [24,25,26]. The solubility of ACV in the ILs and the IL/O MEs, the hydrodynamic size and size distribution of the ME droplets, and the stability of the MEs were studied. In addition, in vitro drug permeation into and across the skin was investigated using Yucatan micro pig (YMP) skin. Finally, a cytotoxicity study of the ILs and the IL/O MEs was performed using a three-dimensional cultured human epidermis model (LabCyte EPI-MODEL) and histological analysis.

## 2. Materials and Methods

### 2.1. Materials

ACV and IPM were obtained from Tokyo Chemical Industries Co. Ltd. (Tokyo, Japan). Choline chloride ([Ch][Cl]), silver oxide (Ag_2_O), lactic acid, oleic acid, methanol, and acetonitrile were purchased from Wako Pure Chemical Industries Ltd. (Osaka, Japan). Formic acid and propionic acid were obtained from Kishida Chemical Co., Ltd., (Osaka, Japan). Tween-80 and Span-20 were procured from Sigma–Aldrich Chemical Co., (St. Louis, MO, USA). The skin of a female Yucatan micropig (YMP) was received from Charles River Japan Inc., Yokohama, Japan.

The three-dimensional cultured human epidermis model (LabCyte EPI-Model 12) was supplied by Japan Tissue Engineering Co., Ltd., Gamagori Miyakitadori, Aichi, Japan. 3-[4,5-Dimethylthiazol-2-yl]-2,5-diphenyltetrazolium bromide (MTT) was obtained from Dojindo Molecular Technologies, Inc., Kumamoto, Japan. All other chemicals and solvents used in the experiments were of analytical grade.

### 2.2. Synthesis of ILs

Biocompatible ILs ([Ch][For], [Ch][Lac], and [Ch][Pro]) and a SAIL ([Ch][Ole]) were selected based on their good toxicity and degradation profiles. The ILs and SAIL were synthesized using a two-step metathesis reaction following an established procedure with slight modification [24,26]. In the first step, choline hydroxide ([Ch][OH]) was synthesized by mixing a predetermined amount of choline chloride ([Ch][Cl]) and an excess amount of Ag_2_O in Milli-Q water (Milli-Q) at room temperature for 2 h. Excess Ag_2_O was removed from the reaction medium by centrifugation and filtration to obtain [Ch][OH]. In the second step, freshly prepared [Ch][OH] was neutralized with an equimolar aqueous solution of carboxylic acid (formic, lactic, or propionic acid) by continuous stirring at room temperature for 24 h (Scheme S1). As oleic acid was not soluble in Milli-Q, the neutralization of [Ch][OH] and oleic acid was performed in methanol instead of Milli-Q [23,26]. The solvent was evaporated using a rotary evaporator (EYELA, NVC-2200, Bohemia, NY, USA) at 40 °C. Finally, the synthesized ILs were freeze-dried for 48 h to evaporate the remaining solvents completely and the reaction yields were ≥85%. The synthesis of the ILs was confirmed by characterization using ^1^H-NMR spectroscopy (JEOL Delta, ECZS NMR spectrometer, 400 MHz, Tokyo, Japan). The water content of all synthesized ILs was determined by Karl Fischer (KF) titration. 

### 2.3. Solubility of ACV in [Ch][CA] ILs

The solubility of ACV in three choline carboxylic acid ILs ([Ch][CA]: [Ch][For]; [Ch][Lac]; and [Ch][Pro]) was determined by adding an excess amount of ACV to the ILs followed by continuous stirring for 24 h at 25 °C. Then, the undissolved ACV was removed by centrifugation followed by filtration using a syringe-driven filter (Millipore, 0.45 μm diameter). IPM and Milli-Q were used as a comparative control instead of the ILs. Finally, the filtrates were analyzed by ultraviolet-visible (UV-vis) spectrophotometry at 252 nm with suitable dilution in methanol to determine the ACV concentration according to a literature procedure [9].

### 2.4. Phase Behavior Studies of IL/S/Co_mix_/IPM Systems: Preparation of IL/O MEs

A phase behavior study was performed prior to ME formation. First, the miscibility of the [Ch][CA] ILs was checked in the [Ch][Ole]/Span-20/IPM systems, where [Ch][Ole] SAIL and Span-20 were used as a surfactant (S) and co-surfactant (Co), respectively. Briefly, S and Co were blended in different weight ratios (*w*/*w*) (1:0, 3:1, 2:1, 3:2, 1:1, 2:3, 1:3, and 0:1). Then, a 15 wt.% of the S and Co mixture (S/Co_mix_) was added to an appropriate amount of IPM, and the mixture was vigorously vortexed to obtain a clear and optically transparent homogeneous solution. Finally, [Ch][CA] ILs were added dropwise individually with continuous stirring until the final mixture turned turbid. The experiment was carried out at room temperature. Then, phase behavior studies were performed as stated above at selected weight ratios (2:1, 3:2, 1:1, 2:3, and 1:3) of S and Co, where the total S/Co_mix_ was maintained at 5 to 75 wt.%. For [Ch][For] and [Ch][Lac] ILs, the experiment was performed at a 2:1 ratio of S/Co_mix_ only. In addition, [Ch][Ole], Tween-80, and Span-20 were also mixed at a 1:1:1 weight ratio where [Ch][Pro] IL was used to carry out further processes.

Finally, IL/O MEs (IL/S/Co_mix_/IPM) were prepared as stated above, where ILs, S/Co_mix_, and IPM were maintained at 3, 15, and 82 wt.%, respectively. A water-in-oil (W/O) ME was also prepared where 3 wt.% Milli-Q was added instead of the IL, using the latter process, as shown in Table 1.

### 2.5. Viscosity, Density, and pH of the ILs and IL/O MEs

The viscosity, density, and pH of each [Ch][CA] IL and IL/O ME formulation were measured at 25 °C using an automated micro-viscometer (Anton Paar Micro-viscometer, c, 2000M/ME, Graz, Austria), micro-densitometer (Anton Paar Density Meter, DMA 35N, Graz, Austria), and pH meter (TOA, HM-30R), respectively. The viscosity of the tested samples was evaluated considering the rolling time of the ball in a sample-filled glass capillary.

### 2.6. Drug Loading Capacity of the IL/O MEs

To determine the maximum drug loading capacity of the MEs, an excess amount of ACV was added to the ME formulations. The ACV-loaded MEs were stirred for 24 h at room temperature. The unloaded ACV was removed using a centrifugation and filtration method. The amount of drug in the subsequent clear filtrate was measured using a UV spectrophotometer as described in Section 2.3.

### 2.7. Particle Size Determination

The hydrodynamic size and polydispersity index (PDI) of drug-loaded/unloaded MEs were determined by dynamic light scattering (DLS: Zetasizer Nano ZS, Malvern Instruments, Worcestershire, United Kingdom). All the tested samples were equilibrated for more than 4 h before starting measurements, and there was no visible macroscopic heterogeneity. Samples were equilibrated for approximately 10 min before collecting data. The average diameters of the tested samples were calculated using five replicated experiments.

### 2.8. Stability of the ME Formulations

The stability of the drug-loaded MEs was investigated for two months considering storage time and storage temperature. The stability was determined by measuring the droplet size using DLS and visual inspection at regular intervals. In addition, the physical stability of the MEs was determined by centrifugation for 30 min at 15,000 rpm. The chemical stability was also examined by measuring the drug degradation extent and encapsulation efficiency. 

### 2.9. Skin Permeation Studies

In vitro drug permeation was investigated on YMP full thickness skin using hand-made Franz diffusion cells (10 mm diameter), consisting of donor and receiver compartments. The prepared skins (2 cm × 2 cm) were soaked in D-PBS solution for 1 h prior to the permeation experiment. The skins were then clamped on the Franz cell with the SC facing up to the donor compartment and the dermis contacting with the receiver phase (D-PBS, pH 7.4). Then 0.5 mL of each of the ACV-loaded formulations (IL/O MEs and controls) were applied on the donor compartment. The receiver compartment was maintained thermostatically at 32.5 ± 0.1 °C using a circulating water bath (NTT-20S, Tokyo Rikakikai Co. Ltd., Tokyo, Japan) and magnetically stirred at 500 rpm during the entire experiment. After a fixed interval, 0.5 mL of the receiver solution was withdrawn to determine the transdermal drug delivery content (permeated across the skin), while an equal amount of fresh D-PBS was added to maintain a constant volume (5 mL) of the receiver solution. After 48 h, the skins were unclamped and washed with 0.1 M HCL three times to remove the tested formulations completely from the skin surface. Finally, the treated skins were processed according to our previous report [27] to estimate the topical drug delivery content (penetrated into the skin). The concentration of ACV was determined using HPLC with a Shiseido CAPCELL PAK C18 MG (4.6 mm × 250 mm) column using the United States Pharmacopeia (USP) method according to a previous report [28], where the mobile phase was 0.02 M glacial acetic acid with elution at a flow rate 1.5 mL/min, and the injected volume of sample was 100 µL. The concentration range of the standard curves was 0–25 µg/mL, and the squared correlation coefficient of the standard curve was more than 0.99 (R^2^ > 0.99). 

### 2.10. Calculation of Skin Permeation Parameters

The cumulative amount of ACV (*Q_h_*, µg/cm^2^) that permeated across the skin was plotted as a function of time, in order to determine the various permeability parameters, where the transdermal flux (*J*, µg/cm^2^/h) was calculated as the slope. The permeability coefficient (*K_P_*, cm/h), was measured using the following equation: *K_P_* = *J*/*C_d_*, where *C_d_* (µg/mL) was the concentration of drug in the donor phase. Lag time (*t_L_*, h) was the intercept of the X-axis. The diffusion coefficient (*D*, cm^2^/h) was calculated from the lag time by the equation, *D* = l2/6*t_L_*, where l (cm) was the thickness of the skin. The skin partition coefficient, (*K_skin_*), was calculated from the following equation: *K_skin_* = *(Jl)/(DC_d_)*. 

### 2.11. Impact of the IL/O MEs on the Skin Barrier Properties

The skin samples were prepared according to a previous report with some modifications [29]. Full thickness YMP skin was thawed and allowed to stand for 1 h at room temperature. Then, the fat portion of the skin was cut off to make it moisture free, and the skin was incubated at 60 °C for 1–2 min to loosen the epidermis. After pulling out the epidermis, the epidermis was floated on a 0.25% trypsin and 1 mM EDTA solution for 24 h at room temperature. The SC side of the epidermis was faced up during floating. Then the SC was isolated from the epidermis and washed with water and allowed to dry for 24 h at room temperature. After cutting into the desired size, the SC was soaked in the test MEs for 30 min at room temperature. Then, the SC was withdrawn from the test samples and washed thoroughly with 20% ethanol and allowed to dry for 1 h. Finally, the treated SC was analyzed by Fourier transform infrared spectroscopy (FTIR) and compared with untreated skin (control).

### 2.12. Cytotoxicity Evaluation of ILs and IL/O MEs

The in vitro cytotoxicity study was performed using a three-dimensional cultured human epidermis model (LabCyte EPI-Model 12, J-TEC, Japan) according to a previous report with some modifications [9]. Briefly, the tissues were cultured into 24-well plates (BD Biosciences, San Jose, CA, USA) with assay medium (0.5 mL) and were incubated for 24 h at 37 °C in a 5% CO_2_ humidified environment. Then, 25 µL of the test formulations were applied into each well on the tissue surface, D-PBS and commercial IL [C1mim][DMP] treated samples were used as negative and positive controls, respectively. The cultures were then incubated for 24 h (37 °C, 5% CO_2_). After that, the tissues were withdrawn from the culture media and washed 15 times with D-PBS carefully to remove any remaining formulation from the tissue surface. Then, 0.5 mL of freshly prepared MTT solution (0.5 mg/mL) was added to each well, and the wells were incubated for 3 h (37 °C, 5% CO_2_). The tissues were then immersed fully into 0.5 mL of propan-2-ol containing micro-tubes and allowed to stand in a refrigerator for 48 h after covering with aluminum foil. Finally, 100 µL of the extracted solutions was transferred into a 96-well plate for measuring the optical density at 570 and 650 nm (as a reference absorbance) using a microplate reader (iMARK, Bio-Rad, Tokyo, Japan), where propon-2-ol was used as a blank. The cell viability was calculated as the percentage relative to the negative control, D-PBS.

### 2.13. Histological Study

The dermal safety of the tested IL/O MEs was investigated on YMP skin according to a previous report [9]. First, the desired size (2 cm × 2 cm) skins were treated with the IL/O MEs or D-PBS (1.0 mL) for 24 h where D-PBS was used as the control. The skin samples were then immersed into Histo Prep compound (Fisher Scientific, Branchburg, NJ, USA) at −80 °C followed by sectioning using a cryostat microtome (CM1510; Leica, Wetzlar, Germany) and placed on glass slides. The slides were then stained with hematoxylin and eosin solution (Muto Pure Chemicals Co. Ltd, Tokyo, Japan). Finally, the specimens were investigated under a high-powered light microscope (BZ-9000 BIOREVO, Keyence Corp., Itasca, IL, USA).

### 2.14. Statistical Data Analysis

The data are given as the mean ± standard deviation (SD). The comparisons between more than two groups were performed by two-way ANOVA analysis for multiple comparison tests using GraphPad Prism software (Version 6.05). The differences were considered significant at *p* < 0.05.

## 3. Results and Discussion

### 3.1. The Solubility of ACV in the [Ch][CA] ILs and Relationship to the Physical Properties of the ILs

The ILs and SAIL used in this study were selected by considering the two important factors, biocompatibility and toxicity. From a structure-activity relationship point of view, choline is the most suitable candidate as a cation, as choline can be derived from various natural sources and has multiple biological functionalities [18,30]. Choline is also known as a source of macronutrients [18,24]. Carboxylic acids are GRAS and have been widely used as pharmaceutical solvents for a long time [25,31,32]. Therefore, three [Ch][CA] ILs consisting of choline as the cation and a carboxylic acid (formic, lactic, or propionic acid) as the anion [Ch][For], [Ch][Lac], and [Ch][Pro], respectively, and one SAIL consisting of choline as the cation and a long chain (C18) fatty acid as the anion [Ch][Ole], were considered to be safe and biocompatible for further study in a TDDS. [Ch][For], [Ch][Lac], and [Ch][Pro] are hydrophilic in nature and intended for use to investigate the solubilization capacity of the sparingly soluble drug, ACV and [Ch][Ole] is known to act as a surfactant [26].

Three fundamental properties of the [Ch][CA] ILs, the viscosity, density, and pH were measured, as shown in Table 2. The viscosity and density of the three [Ch][CA] ILs were significantly different with a good agreement to previous reports [24]. The measured pH values varied from 5.5 to 7.6. It has been reported that the physical properties of synthesized ILs are directly influenced by the structure, symmetry, and alkyl chain length of the carboxylic acid [24]. However, the actual relationship of the physical properties of ILs with the nature of the cation/anions could not be established in this study.

The maximum solubility of ACV in the ILs was 203, 208, and 278 mg/mL for [Ch][For], [Ch][Lac], and [Ch][Pro], respectively, which was significantly higher compared with Milli-Q and IPM (Table 2). As the ILs were hydrophilic in nature with strong H-bond accepting anions, ACV may be dissolved in these ILs through the formation of H-bonds, van der Waals forces, or π–π interactions between the polar groups of the drug and the IL anions [8]. It has been reported that the solubility of a drug depends on several factors, i.e., H-bond accepting ability, density, viscosity, and the alkyl/aromatic side chains of the IL anion [33,34]. The solubility of ACV was higher in [Ch][Pro] owing to its low viscosity, and the small charge-localized anion. On the other hand, [Ch][For] formed interionic hydrogen bonds, having a very small charge-localized anion and [Ch][Lac] formed intramolecular hydrogen bonds, having an extra –OH group, resulting in less hydrogen bonding ability with ACV and less ACV solubilizing capacity [33].

### 3.2. Phase Behavior Studies of IL/S/Co_mix_/IPM Systems: Preparation of IL/O MEs

**Figure 1:**
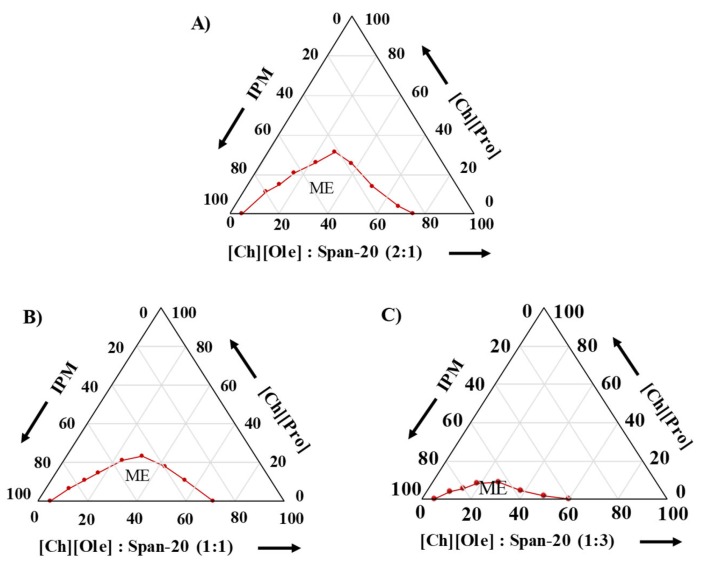
Phase behavior studies of IL/S/Co_mix_/IPM MEs consisting of [Ch][Pro] with varying weight ratios of S/Co (**A**) 2:1, (**B**) 1:1, and (**C**) 1:3 at 25 °C.

A phase behavior study is very important for selecting the optimum composition of the MEs. Prior to the phase behavior study, a miscibility study of the ILs in a S/Co_mix_/IPM system (consisting of 15 wt.% S/Co_mix_ at different S/Co weight ratios) was performed, and it was found that a higher [Ch][Ole] content in the S/Co_mix_ was favorable for the miscibility of all ILs in S/Co_mix_/IPM system, as shown in Figure S1. However, all the ILs were immiscible at 0:1, 1:0, and 3:1 S/Co ratios in S/Co_mix_/IPM and the miscibility of [Ch][For] and [Ch][Lac] was very low at all S/Co ratios, except 2:1. Therefore, the phase behavior study was performed at 2:1, 3:2; 1:1, 2:3, and 1:3 S/Co ratios for [Ch][Pro] (Figure 1 and Figure S2), whereas only a 2:1 ratio was used for [Ch][For] and [Ch][Lac] (Figure S3). In the case of [Ch][Pro], it was found that the area of the single phase or ME forming regions varied with the S/Co ratio and the trend was 2:1 > 3:2 > 1:1 > 2:3 > 1.3 (Figure 1 and Figure S2). As the ILs are hydrophilic in nature and immiscible with IPM, they must be located in the core of the micelle owing to the hydrogen bond formation between the –OH groups of S/Co and the anions of the ILs, and the strong electrostatic interaction between the positive head group of Ch][Ole] and the anion of the ILs [8]. Because of the strong electrostatic interaction between the head group of [Ch][Ole] and the anion of the ILs, a higher content of [Ch][Ole] in the S/Co_mix_ favored ME formation [9,35]. To compare the surface activity of [Ch][Ole] and Tween-80, the phase behavior of a ME consisting of [Ch][Ole]/Tween-80/Span-20 at a 1:1:1 weight ratio was studied (Figure S2), and it was found that replacing [Ch][Ole] by the same amount of Tween-80 caused the ME to lose IL holding capacity. This result indicated that [Ch][Ole] has a higher surface activity than Tween-80, owing to the higher electrostatic interaction between the head group of [Ch][Ole] and the anion of the polar IL [9]. In addition, compared with conventional surfactants, a much lower percentage of S/Co_mix_ was required to solubilize a large amount of ILs using [Ch][Ole]. It has been reported that MEs required a comparatively reduced amount of surfactant with a two or more surfactant mixture than with a single surfactant [36]. This interesting finding can be explained in terms of favorable interfacial properties (e.g., rigidity and polarity) provided by the mixture of the two surfactants [8]. Among the three ILs, [Ch][Pro] had the maximum ME formation capacity, which was indicated by the larger single-phase area in the phase diagram (Figure S3) and this greater capacity was because of the greater hydrogen bonding ability of [Ch][Pro] [33].

Finally, we prepared MEs with optimum compositions (Table 1). Though a larger amount of S/Co_mix_ in the MEs could retain a larger amount of ILs, resulting in a larger amount of drug that could be loaded, this reduced the permeability [13] and increased the toxicity [14]. Therefore, we selected a comparatively lower content of IL and S/Co_mix_, and a higher content of IPM in the ME formulations in this study. ME6 was prepared to compare the surface activity, drug loading capacity, permeability, and toxicity of [Ch][Ole] and Tween-80.

### 3.3. Density and Viscosity of the MEs

The density of the MEs decreased with increasing [Ch][Ole] content in the S/Co_mix_ because of the lower density of [Ch][Ole] (0.98 g/cm^3^) compared with Span-20 (1.032 g/cm^3^), but the differences were not significant among them. The viscosity of the MEs varied significantly with the S/Co ratio and increased with increasing [Ch][Ole] content in the S/Co_mix_, whereas the viscosity did not depend on the ILs, Table S1.

### 3.4. Particle Size Determination

The hydrodynamic size and PDI of the MEs were determined by DLS. The particle size variation of the MEs (consisting of 15 wt.% S/Co_mix_ at a 2:1 weight ratio), with varying R values (molar ratios of IL per S/Co_mix_) were studied, and it was found that the particle size increased with increasing R values, which confirmed that the IL was located in the hydrophilic micelle core (Figure S4). When the IL was added, the additional IL entered the core of the ME. To cover the additional IL, the surfactant aggregates were expanded, resulting in a larger particle diameter of the MEs [8]. In addition, by plotting particle size as a function of R, it was found that the particle size was an almost linear function of R, as shown in Figure S5, and according to the swelling law of MEs, this indicated spherical ME droplets [37]. Moreover, the particle size of the MEs was also studied at constant R values (by varying the surfactant and IL), and it was found that with increasing surfactant content, the number of particles was also increased while keeping a constant particle size (Figure S6) [8].

**Figure 2:**
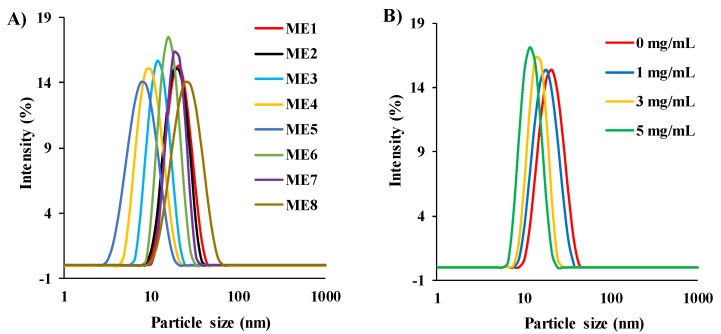
The size and size distribution of (**A**) drug-free MEs with varying S/Co weight ratios and (**B**) drug-loaded (0–5 mg/mL) ME1 at 25 °C.

The particle size of the MEs was determined with varying S/Co weight ratios, and it was found that the particle size of the MEs varied from 17.7 to 31.3 nm (less than 100 nm), which indicated satisfactory MEs [38], and the particle size increased with increasing [Ch][Ole] and/or decreasing Span-20 content (Figure 2A). This trend can be explained based on the content of the individual surfactants in the S/Co_mix_. With higher [Ch][Ole] content in the S/Co_mix_, the head group of [Ch][Ole] might face steric hindrance with the cation of the polar IL, and consequently, the particle size increased. On the other hand, with a higher content of Span-20, the ME has the ability to form smaller reverse micelles in the organic media by marked surface bending of the large hydrophobic chains [10,39]. In addition, there is a positive correlation between the viscosity and the particle size of MEs [9,10]. The particle size of the [Ch][Pro]-based ME was smaller than [Ch][For] and [Ch][Lac] at the same S/Co ratio owing to the stronger hydrogen bonding ability of [Ch][Pro] (Figure 2A).

The size and size distribution of ACV-loaded (2 mg/mL) MEs with varying S/Co ratios were also studied to investigate the effect of drug loading on the droplet size, and it was found that the particle size decreased compared with the drug-free MEs for all S/Co ratios studied (Figure S7). In further investigations, the particle size of ME1, loaded with different concentrations (0, 1, 3, and 5 mg/mL) of ACV was also studied, and it was found that the particle size decreased from 31 to 22 nm with increasing drug concentration (Figure 2B), and this trend was in good agreement with previous literature [9,40]. There may be two possible reasons for this effect, firstly, at higher drug concentrations, a certain amount of drug might be deposited into the interphase of the ME, and thus, reduce the particle size by acting as an emulsifying agent and secondly, the deposited drug at the interphase could reduce the surfactant movement and consequently reduce the particle size [9]. The small values of the PDI (<0.3) indicated the homogeneity of the prepared MEs [38].

### 3.5. Drug Loading Capacity of the IL/O MEs

**Figure 3:**
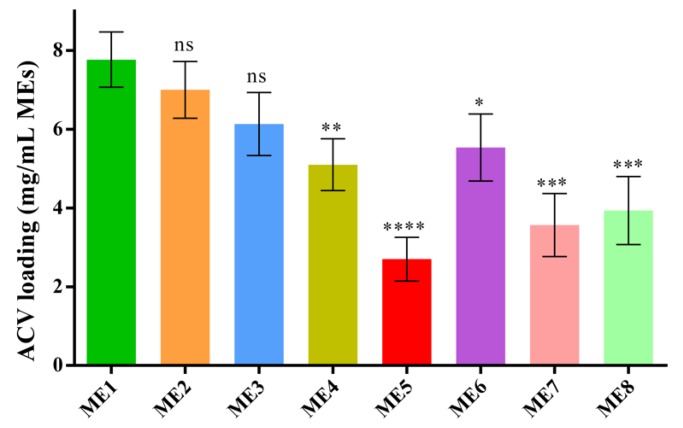
ACV-loading capacity of MEs at 25 °C; (mean ± SD, *n* = 3, ns: not significant, * *p* < 0.05, ** *p* < 0.01, *** *p* < 0.001, and **** *p* < 0.0001 using Dunnett’s multiple comparison test.

The drug loading capacity of the IL/O MEs was estimated to assess them as a vehicle for the delivery of a sparingly soluble drug. Though the ACV-loading capacity of the IL-free S/Co_mix_/IPM system was very low (0.15 mg/mL for S/Co = 2:1, 15 wt.%), the capacity was increased dramatically by incorporating IL into this system (7.7 mg/mL for ME1). The ACV-loading capacity was decreased with decreasing [Ch][Ole] content in the S/Co_mix_, and it was significantly decreased when [Ch][Ole] < Span-20 in the S/Co_mix_, as shown in Figure 3. The ACV-loading capacity also depended on the type of IL. Comparing ME1, ME7, and ME8, it was found that ME1 had a significantly higher loading capacity than ME7 or ME8, owing to the higher ACV solubilizing capacity of [Ch][Pro]. It has been reported that the drug loading capacity of IL/O MEs highly depends on the categories of IL [10]. In addition, comparing ME1 and ME6, it can be seen that [Ch][Ole] had more influence on the drug loading in this system than Tween-80. Among all the formulations, ME1 showed the highest drug loading capacity because of its larger stable interface compared with the other MEs [9,10,41]. Moreover, the relative solubility of the drug in [Ch][Ole], Tween-80, and Span-20 would have a contribution to the ability of a given ME to entrap the drug [9].

### 3.6. Stability of the Drug-Loaded MEs

**Figure 4:**
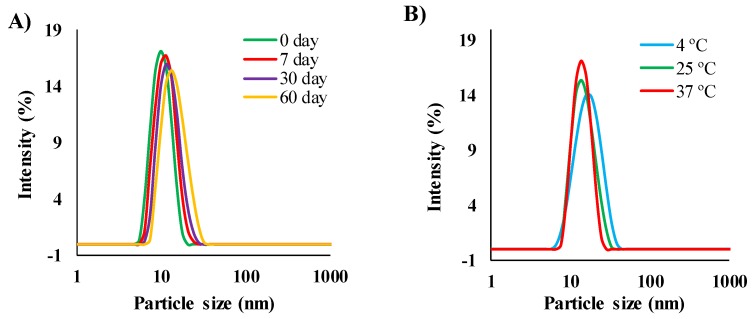
The size and size distribution of ACV-loaded ME1 (**A**) effect of storage time at 25 °C and (**B**) effect of storage temperature after two months.

It is important to note that MEs need to be stable to be used as drug delivery carriers. To investigate the stability of the MEs, in this study we examined ACV (5 mg/mL) loaded MEs (ME1, ME2, ME3, ME4, and ME6) over two months at 25 °C. As the ACV-loading capacities of ME5, ME7, and ME8 were <5 mg/mL, they were not considered for stability and drug delivery experiments. No significant change was found in terms of clarity and phase separation observations, and the particle sizes of ME1 and ME2, during the entire observation time. The particle size of ME1 was increased slightly from 22 to 25 nm, which was not significant, as shown in Figure 4A. However, the particle sizes of ME3, ME4, and ME6 started to increase linearly from 30 days, and finally, the samples became turbid after 45 days, which confirmed the formation of stable MEs with a higher [Ch][Ole] content in the S/Co_mix_. It has been reported that SAIL can increase the stability of MEs [15,16]. No physical instabilities (e.g., phase separation, phase inversion, aggregation, or cracking) of the MEs were found by centrifugation, which confirmed the physical stability and excellent drug encapsulation efficiency of the MEs [42]. In addition, ACV-loaded ME1 and ME2 were stored at different temperatures (4, 25, and 37 °C) for two months to assess the effect of storage temperature on stability. After several time intervals, the MEs were examined by visual inspection and particle size determination. No significant change was found for either ME after two months (Figure 4B for ME1), indicating a negligible impact of the storage temperature on the long-term stability of drug-loaded MEs. This stability can be explained in terms of the ionic ([Ch][Ole]) and non-ionic (Span-20) character of the surfactant. It has been reported that a mixture of ionic and non-ionic surfactants can form temperature-insensitive MEs owing to their synergistic effects [36]. The chemical stability of ACV-loaded ME1 and ME2 formulations was investigated using HPLC, and it was found that the MEs showed excellent encapsulation efficiency of ACV. After two months, the encapsulation efficiency of both MEs (contained 5 mg/mL ACV initially) was ≥98%, indicating no degradation (Figure S8).

### 3.7. Skin Permeation Studies

**Figure 5:**
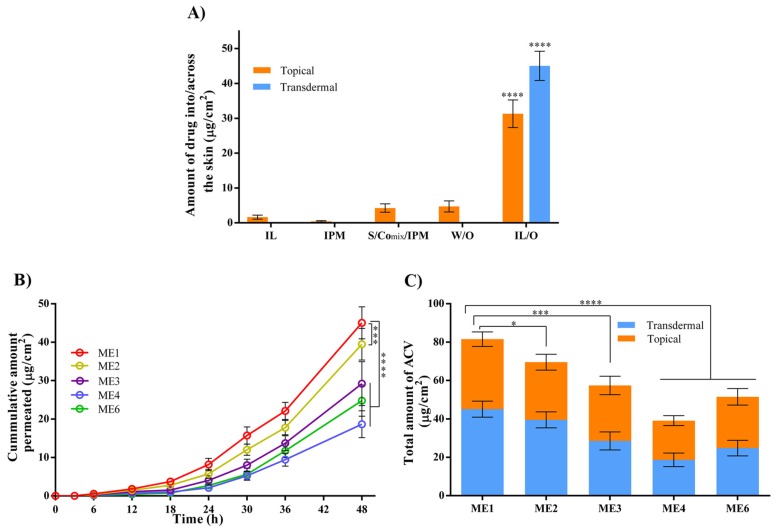
(**A**) Topical and transdermal delivery of ACV from various drug carriers after 48 h, where S/Co_mix_/IPM: 15 wt.% S/Co_mix_ at a 2:1 ratio in IPM. W/O: ME9. IL/O: ME1; (**B**) transdermal permeation profile of ACV from various IL/O MEs with varying S/Co ratios; (**C**) the total (topical + transdermal) delivery of ACV from various IL/O MEs with varying S/Co ratios after 48 h; (mean ± SD, *n* = 3, * *p* < 0.05, *** *p* < 0.001, and **** *p* < 0.0001 using Dunnett’s multiple comparison test. All the drug carriers contained 5 mg/mL ACV.

In vitro drug permeation studies were performed using YMP skin owing to its similar clinical, structural, and immuno-histochemical features to human skin [9]. First, the topical and transdermal delivery of ACV from IL/O MEs was investigated and compared with other formulations (Figure 5A). The topical delivery of ACV from IPM, S/Co_mix_/IPM, and W/O MEs was very low, while the transdermal delivery was below the detection limit. Interestingly, compared with the other formulations, the IL/O ME demonstrated significantly enhanced topical and transdermal delivery with values of 36.47 and 45.05 µg/cm^2^, respectively. This dramatically enhanced permeation using the IL/O MEs was found because of their high drug solubilizing capacity and promising drug conveyances technique. Generally, drugs are administrated into the skin in a solubilized state. A large amount of drug was loaded into the core of the IL/O ME solubilized by IL, which could act as a drug reservoir and provide a greater concentration gradient to the skin [9]. Whereas, IPM (a potential enhancer) disrupts the barrier function of the skin, which facilitated to enter the nano-sized drug-loaded IL droplets into the skin [11]. Nonetheless, ACV was solubilized state in IL, but as it is hydrophilic in nature, IL alone could not deliver ACV due to of the strong hydrophobic barrier functions of the skin [9]. On the other hand, though IPM, S/Comix/IPM, and W/O MEs disrupt the barrier function as they contain IPM, ACV could not permeate across the skin from these formulations because ACV was suspended in these systems, which probably obstructed the access of ACV to the skin [9,28].

It has been reported that the molar ratio of individual surfactants can influence drug delivery by controlling the physicochemical properties of the MEs [43]. Therefore, the delivery of ACV from various MEs with varying S/Co ratios was studied. From the cumulative permeation profiles (Figure 5B), it can be seen that ME1 enhanced the transdermal delivery significantly compared with the other MEs. Other permeation parameters, including transdermal flux, permeability coefficient, diffusion coefficient, and skin partition coefficient were determined from the cumulative permeation profile. It was found that all these parameters were increased with increasing [Ch][Ole] content in the MEs, as shown in Table 3, indicating that higher [Ch][Ole] content was favored for transdermal delivery. In fact, the transdermal delivery of drug mainly depends on the transdermal flux and permeation coefficient. The highest transdermal flux (1.43 µg/cm^2^/h) and permeation coefficient (2.86 × 10^−4^ cm/h) were both found for ME1, because of the higher skin partition and diffusion coefficients (indicating better solvent distribution ability into the deeper layers of the skin) resulting in ME1 having the highest transdermal delivery of ACV [11,44].

In addition, the topical delivery of ACV was investigated. The total (topical and transdermal) delivery after 48 h is presented in Figure 5C. It was observed that, as for transdermal delivery, topical delivery was also favored using ME1, having a higher [Ch][Ole] content, resulting in the highest drug delivery. This result could be explained based on the higher interfacial area and stability of ME1. It has been reported that a larger stable interfacial area of ME droplets favors transdermal and topical delivery [9,45]. Comparing the transdermal delivery between ME1 and ME6, it was revealed that [Ch][Ole] significantly enhanced the permeability compared with Tween-80 (Table 3). In addition, by comparison with a previous report [9], (where a very low amount of ACV was permeated using a Tween-80/Span-20 surfactant-based ME, using the same experimental protocol), we can claim that the [Ch][Ole] has a significantly greater permeation enhancing ability compared with Tween-80. This enhanced ability can be explained in terms of the influence of the [Ch][Ole] on the skin modification being ionic in character [11]. Hence, the effect of IL/O MEs on the skin barrier properties was further studied. 

As ME1 displayed the significantly higher transdermal flux and permeation coefficient (Table 3), and delivered the significantly higher amount of drug topically and transdermally (Figure 5C) than other MEs, could be the most suitable nano-carrier. 

### 3.8. Impact of IL/O MEs on the Skin Barrier Properties

**Figure 6:**
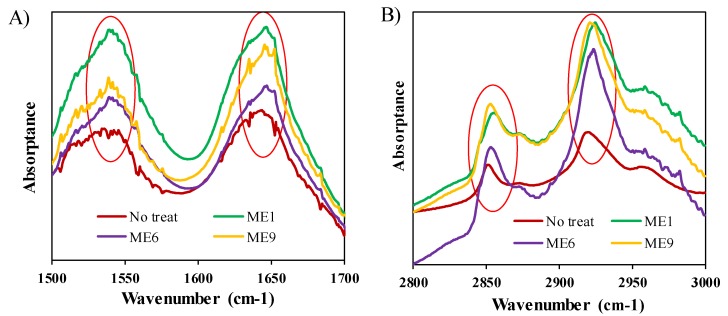
FTIR spectra of (**A**) keratin and (**B**) lipid of SC samples after treatment with different formulations.

FTIR spectroscopy, which can provide deep insight into the molecular structure of the lipid matrix of the SC [46], was performed to assess the structural changes of the SC. To avoid the interference of ACV, drug-free MEs were applied, and the results were compared with an untreated sample (as control), as shown in Figure 6 and Table S2. All the treated samples produced some red shifts of the absorption peaks to higher wavenumber for both the lipid and keratin of the SC. For ME1, the C–H vibration peaks shifted to 2924.5 from 2920 cm^−1^ (asymmetric vibration), and 2854.5 from 2851 cm^−1^ (symmetric vibration), and the NH–C=O vibration peaks shifted to 1647.5 from 16440, and 1540.25 from 1538 cm^−1^. These shifts are directly related to the molecular structure of the skin [46]. When the skin was treated with the MEs, the orthorhombic conformation of the lipids was transformed to a liquid crystalline conformation resulting in the CH_2_ symmetric stretching vibration peak shifting to higher wavenumber. The shift of the NH–C=O vibration peaks to higher wavenumber revealed that the conformation of keratin was converted from an organized α-helical structure to a randomly coiled structure by treatment with the test systems. These shifts occurred because of the presence of lipophilic IPM in the test formulations, which disrupted the barrier function of the skin [11,44]. ME1 had a greater effect on the structural changes of the skin compared with the other MEs in the following order: ME1 ˃ ME6 ˃ ME9, which can be ascribed to the ionic character of the IL [11]. In ME1, the total ionic surfactant ([Ch][Ole]) was 10 wt.%, whereas it was only 5 wt.% in ME6, and ME9 was fully composed of non-ionic surfactants (Tween-80 and Span-20) and had no IL content. It has been reported that MEs containing an IL as surfactant disrupt the barrier function effectively because of their ionic character [11]. It has also been reported that an IL can alter the SC structure transiently by extracting the lipid from the skin [25]. The aforementioned results demonstrate that the IL/O MEs reduced the barrier properties of skin, leading to a high permeability coefficient and good drug delivery [11,47].

### 3.9. Cytotoxicity Evaluation of ILs and IL/O MEs

**Figure 7:**
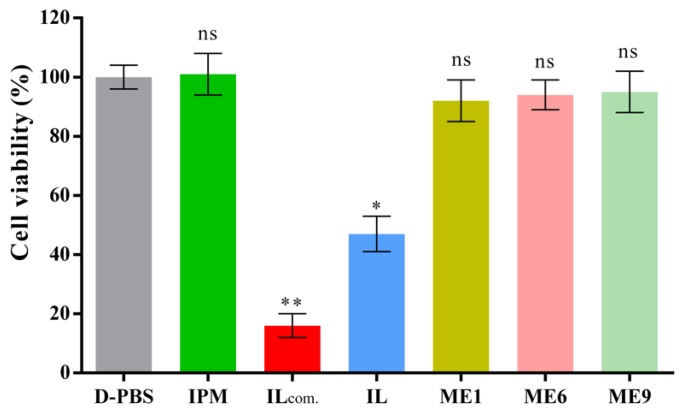
Cytotoxicity evaluation of ILs and MEs using reconstructed human epidermal model LabCyte EPI-MODEL-12 (mean ± SD, *n* = 3), ns: not significant, * *p* < 0.05 and ** *p* < 0.01 using Dunnett’s multiple comparisons test.

In vitro cytotoxicity studies (skin irritation profiles) of the ILs and IL/O MEs were performed using a reconstructed human epidermal model (LabCyte EPI-MODEL-12). In this experiment, [Ch][Pro], ME1, ME6, and ME9 were selected to compare the relative toxicity with D-PBS (as a negative control) and [C1mim][DMP] (a commercial IL: IL_com_ as a positive control), as shown in Figure 7. The cell viability of all the MEs was above 92% compared with D-PBS, and the values were not significantly differed from D-PBS and IPM, which indicated that the prepared IL/O MEs were non-toxic. Comparing between ME1, ME6, and ME9, it was found that the toxicity profile of [Ch][Ole] was similar to Tween-80, which was in a good agreement with a previous report [26]. However, when [Ch][Pro] was used alone, the cell viability decreased to 48%, but the cell viability was below 15% for [C1mim][DMP], which suggested that [Ch][Pro] was less toxic than [C1mim][DMP] IL. The results were in a good agreement with published reports in which choline-based ILs demonstrated less irritation towards different cultured cell lines, such as human keratinocytes cell line (HaCat) [21], human embryonic kidney cell line (HEK-293) [18], and human epidermal keratinocytes-adult cell line (HEK-a) [25]. It has also been reported that imidazolium-based ILs are considered as toxic and less biodegradable, whereas choline-based ILs are regarded as safe, non-toxic and biocompatible [10,20]. This reduced toxicity can be attributed to the biocompatible sources of the cation and anion of [Ch][Pro], choline is used as a food additive and known to be non-toxic and biocompatible [18,25,48], and propionic acid is GRAS and used as preservative in food, cosmetic and pharmaceutical industries [31,32]. Our IL/O MEs, containing a low amount of IL, appears to be non-toxic and are potential carriers for TDDSs.

### 3.10. Histological Study

**Figure 8:**
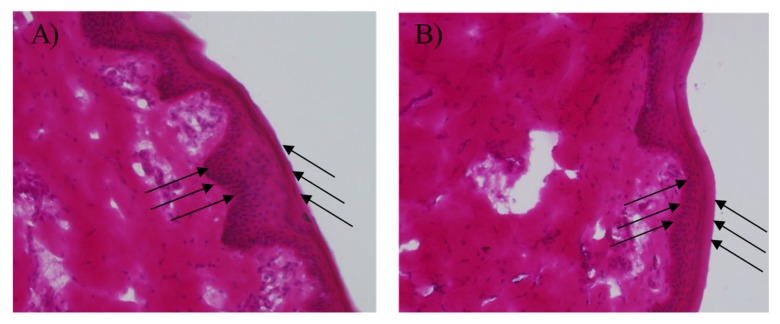
In vitro histopathological evaluation of YMP skin sections (×20) treated with (**A**) D-PBS and (**B**) ME1. The left direction arrows indicate the SC layer and the right direction arrows indicate the epidermis layer.

IL/O MEs need to be safe and non-toxic to be used as transdermal carriers. According to the drug loading capacity, stability, and drug permeation studies, ME1 was the most suitable candidate for use as a transdermal carrier. Therefore, an in vitro histological study was performed to investigate the dermal safety of ME1. The ME1-treated skins were observed through a fluorescence microscope (20-fold magnification), and it was found that the structures of the SC, epidermis, and dermis of the skin were clearly visible and organized after treatment with ME1 for 24 h compared with control (D-PBS treated sample), as shown in Figure 8 which were in a good agreement with a published report [9]. Therefore, the IL/O MEs used in this study had no antagonistic effect on the skin, and could be a safe and promising nano-carrier for the transdermal delivery of sparingly soluble drugs. 

## 4. Conclusions

This study presents a novel IL/O ME which was developed using biocompatible ILs as the non-aqueous polar phase (core of the ME), as well as the surfactant, for an improved TDDS for the sparingly soluble drug, ACV. Preliminary results clearly indicated the optimum S/Co_mix_ in which to prepare a thermodynamically stable ME, with a high drug loading ability and enhanced drug permeability. FTIR investigations revealed that the enhanced drug permeation with the IL/O ME was because of a reduction of skin barrier function via modification and disruption of the regular arrangement of the corneocytes of the SC. If considering the drug loading capacity and skin permeation studies, the successful formation of an ME with [Ch][Pro] in the core as a non-aqueous polar phase could be attributed to the favorable interfacial properties provided by a blend of [Ch][Ole] and Span-20, compared with a blend of Tween-80 and Span-20. Moreover, in vitro skin irritation and histological tests confirmed that the prepared IL/O MEs were safe and non-toxic. Both the ILs, as well as the prepared IL/O MEs, were completely biocompatible, and are potential candidates for future applications in pharmaceutical formulations of sparingly soluble drugs, as well as proteins, peptides, and genetic material, through a TDDS.

## Figures and Tables

**Table 1:** Contents of the microemulsion (ME) formulations ^a^.

Formulations	ILs	Surfactant: Co-Surfactant (Weight Ratio)		
		Surfactant		Co-Surfactant
		[Ch][Ole]	Tween-80	Span-20
ME1	[Ch][Pro]	2	-	1
ME2	[Ch][Pro]	3	-	2
ME3	[Ch][Pro]	1	-	1
ME4	[Ch][Pro]	2	-	3
ME5	[Ch][Pro]	1	-	3
ME6	[Ch][Pro]	1	1	1
ME7	[Ch][For]	2	-	1
ME8	[Ch][Lac]	2	-	1
ME9 ^b^	Milli-Q	-	2	1

^a^ MEs were prepared with overall 15 wt.% S/Co_mix_ and 3 wt.% [Ch][CA] ionic liquid (IL) in isopropyl myristate (IPM). ^b^ ME9 was set as control where 3 wt.% Milli-Q was used as a replacement for [Ch][CA] IL.

**Table 2:** The solubility of acyclovir (ACV) in the [Ch][CA] ILs ^a^ at 25 °C and the effects of the viscosity, density, pH, and anionic domain on IL-mediated dissolution ^b^.

IL or Solvent	Anionic Structure 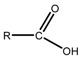	Solubility of ACV (mg/mL)	*ρ* (g/cm^3^)	*η* (m Pa s)	pH
[Ch][For]	R = -H	203 ± 12 ****	1.12 ± 0.03	124.7 ± 7	5.5
[Ch][Lac]	R = -C_2_H_5_O	208 ± 15 ****	1.15 ± 0.02	897.2 ± 27	7.6
[Ch][Pro]	R = -C_2_H_5_	278 ± 18 **^,^****	1.07 ± 0.02 ^†^	309.5 ± 12 ^####^	6.2
IPM	-	0.03 ± 0.01	-	-	-
Milli-Q	-	0.41 ± 0.08	-	-	-

^a^ The solubility of ACV in water and IPM is also given. ^b^ Data are shown as mean ± SD (n = 3). **** compared with Milli-Q and IPM, *p* < 0.0001; ** compared with [Ch][For] and [Ch][Lac], *p* < 0.01; ^†^ compared with [Ch][For] and [Ch][Lac, *p* < 0.05; ^####^ compared with [Ch][For] and [Ch][Lac], *p* < 0.0001 using Sidak’s multiple comparison test.

**Table 3:** Effect of [Ch][Ole] on permeation parameters. Data are shown as mean ± SD, n = 3.

Formu-Lations	Cumulative Amount, *Q_48h_* (µg/cm^2^)	Transdermal Flux, *J* (µg/cm^2^/h)	Permeability Coefficient, *K_P_* (×10^−4^ cm/h)	Diffusion Coefficient, *D* (×10^−4^ cm^2^/h)	Skin Partition Coefficient, *K_Skin_*
ME1	45.05 ± 4.18	1.43 ± 0.13 ***^,^****	2.86 ± 0.24 ***^,^****	2.77 ± 0.22	0.18 ± 0.03
ME2	39.48 ± 4.14	1.21 ± 0.11	2.42 ± 0.21	2.68 ± 0.23	0.16 ± 0.04
ME3	28.53 ± 4.68	0.92 ± 0.11	1.85 ± 0.18	2.63 ± 0.25	0.12 ± 0.03
ME4	18.66 ± 3.50	0.62 ± 0.09	1.23 ± 0.15	2.58 ± 0.17	0.08 ± 0.02
ME6	24.78 ± 4.05	0.83 ± 0.1	1.66 ± 0.18	2.48 ± 0.22	0.12 ± 0.03

*** *p* < 0.001 compared with ME2 and **** *p* < 0.0001 compared with other MEs using Dunnett’s multiple comparisons test.

## Supplementary Materials

The following are available online at https://www.mdpi.com/1999-4923/12/4/392/s1, Figure S1: Effect of [Ch][Ole] content at a fixed S/Co_mix_ concentration (15 wt.%) on the miscibility of [Ch][CA] ILs in a S/Co_mix_/IPM system at 25 °C; (mean ± SD, n = 3). Figure S2. Phase behavior studies of IL/S/Co_mix_/IPM MEs consisting of [Ch][Pro] with varying S/Co weight ratios (A) 3:2 (B) 2:3, and (C) 1:1:1 ([Ch][Ole]: Tween-80: Span-20) at 25 °C. Figure S3. Phase behavior studies of IL/S/Co_mix_/IPM MEs consisting of (A) [Ch][Pro], (B) [Ch][For], and (C) [Ch][Lac] at a 2:1 weight ratio of S/Co at 25 °C. Figure S4. The size and size distribution of IL/S/Co_mix_/IPM ME (consisting of 15 wt.% S/Co_mix_, at a 2:1 weight ratio) with different R values (R = molar ratio of IL and S/Co_mix_) at 25 °C. Figure S5. Dependence of the diameter of IL/S/Co_mix_/IPM ME (consisting of 15 wt.% S/Co_mix_, at a 2:1 weight ratio) on R (R = molar ratio of IL and S/Co_mix_) at 25 °C. Figure S6. The size and size distribution of IL/S/Co_mix_/IPM ME with different S/Co_mix_ concentrations (wt.%) at a 2:1 weight ratio and R = 0.2 at 25 °C. Figure S7. The size and size distribution of ACV-loaded (2 mg/mL) MEs with varying S/Co weight ratios at 25 °C. Figure S8. ACV encapsulation efficiency of MEs after two months. Table 1S. The density and viscosity of MEs. Table S2. FTIR peak shifts of SC after treatment with different MEs (mean ± SD, n = 3).
