# Supplementary material for: Ionic Liquid-In-Oil Microemulsions Prepared with Biocompatible Choline Carboxylic Acids for Improving the Transdermal Delivery of a Sparingly Soluble Drug
Source: Pharmaceutics. 2020 Apr 24;12(4):392. doi: 10.3390/pharmaceutics12040392 (PMC7238071; doi:10.3390/pharmaceutics12040392)
Supplement: Supplementary file 1 [file pharmaceutics-12-00392-s001.pdf]

pharmaceutics

Supplementary Materials: Ionic Liquid-in-Oil Microemulsions Prepared with Biocompatible Choline Carboxylic Acids for Improving the Transdermal Delivery of a Sparingly Soluble Drug

Md. Rafiqul Islam, Md. Raihan Chowdhury, Rie Wakabayashi, Noriho Kamiya, Muhammad Moniruzzaman and Masahiro Goto

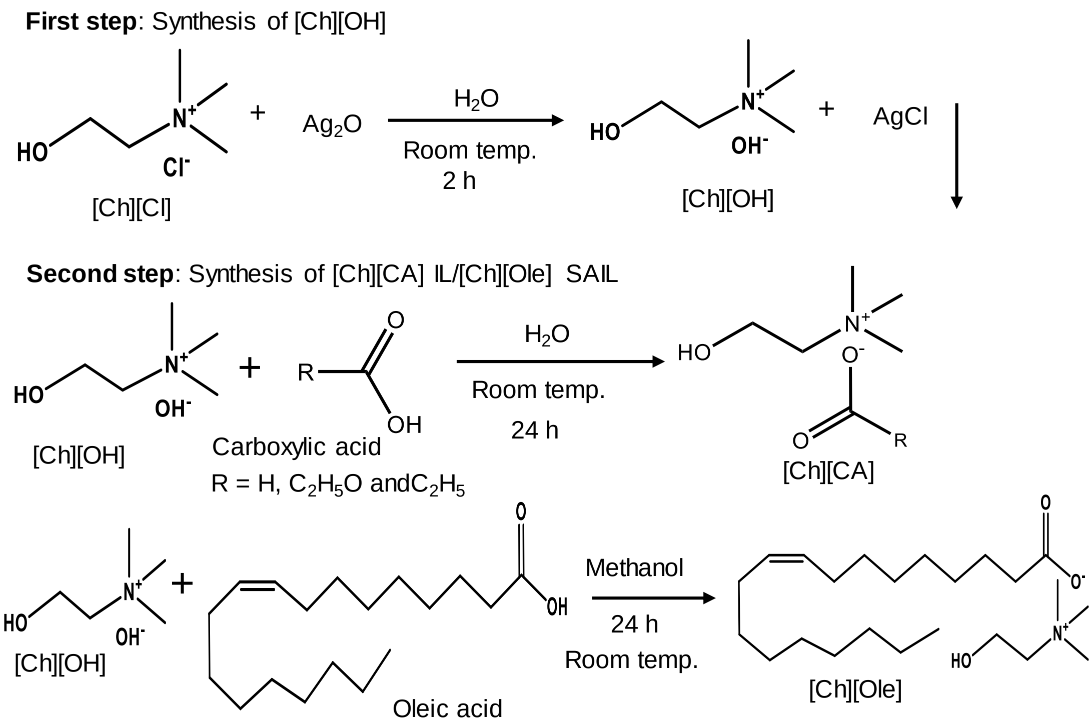

Scheme S1. General process for the synthesis of [Ch][CA] ILs and [Ch][Ole] SAIL.

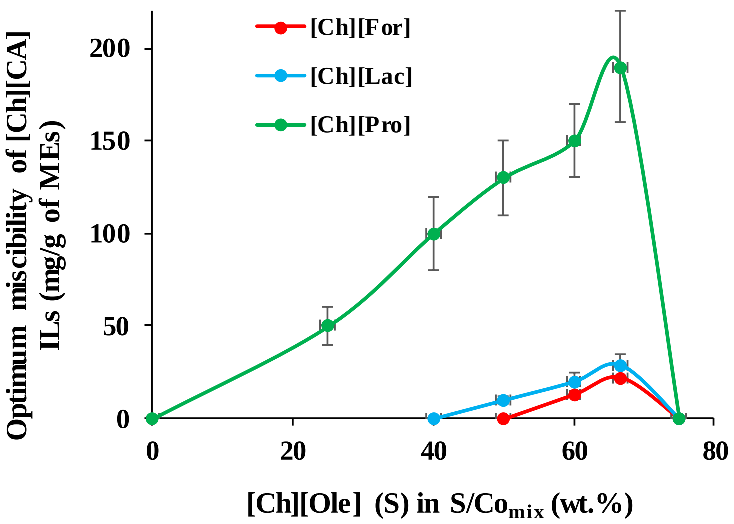

Figure S1. Effect of [Ch][Ole] content at a fixed S/Co$_{mix}$ concentration (15 wt.%) on the miscibility of [Ch][CA] ILs in a S/Co$_{mix}$/IPM system at 25 °C; (mean ± SD, n = 3)

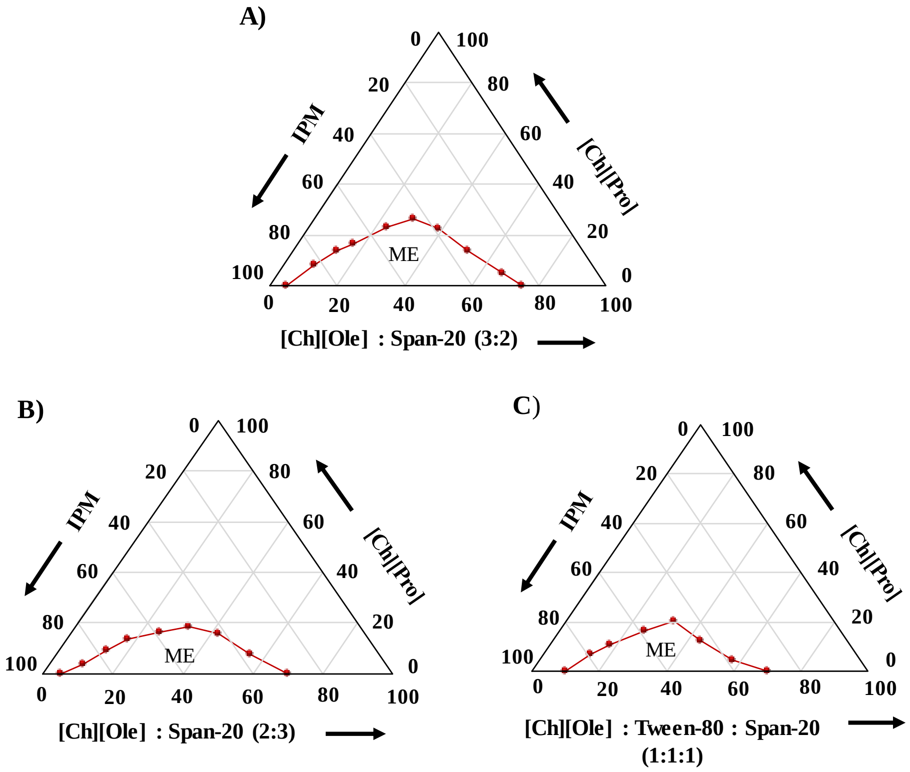

Figure S2. Phase behavior studies of IL/S/Co$_{mix}$/IPM MEs consisting of [Ch][Pro] with varying S/Co weight ratios (A) 3:2 (B) 2:3, and (C) 1:1:1 ([Ch][Ole]: Tween-80: Span-20) at 25 °C.

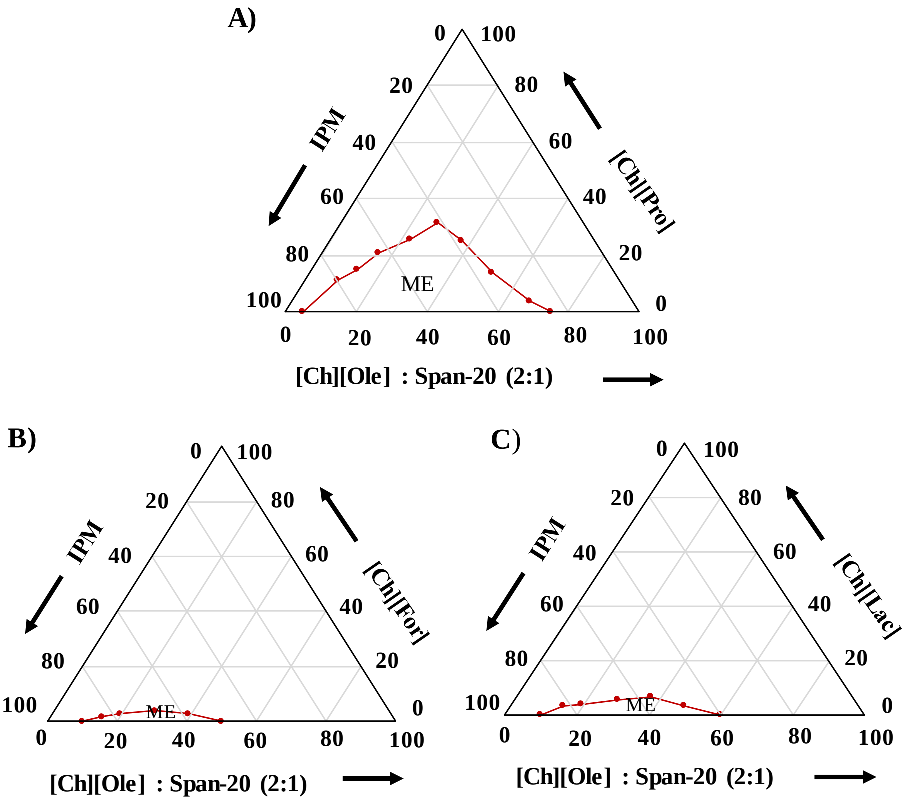

Figure S3. Phase behavior studies of IL/S/Co_{mix}/IPM MEs consisting of (A) [Ch][Pro], (B) [Ch][For], and (C) [Ch][Lac] at a 2:1 weight ratio of S/Co at 25 °C.

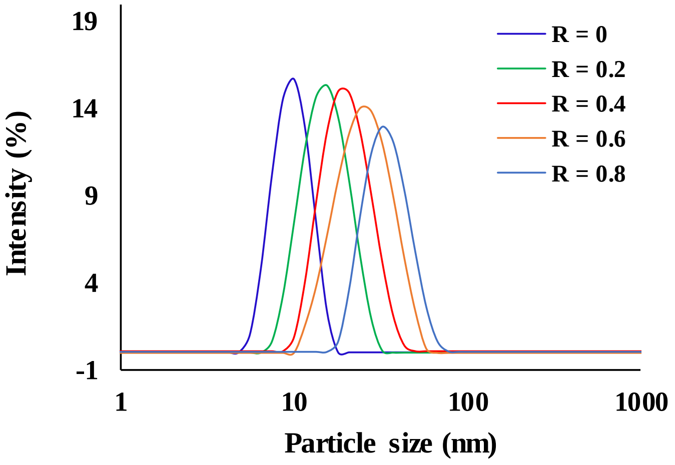

Figure S4. The size and size distribution of IL/S/Co_{mix}/IPM ME (consisting of 15 wt.% S/Co_{mix}, at a 2:1 weight ratio) with different R values (R = molar ratio of IL and S/Co_{mix}) at 25 °C.

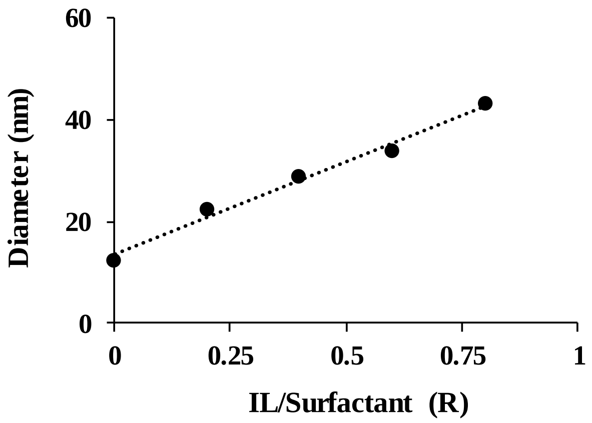

Figure S5. Dependence of the diameter of IL/S/Co_{mix}/IPM ME (consisting of 15 wt.% S/Co_{mix}, at a 2:1 weight ratio) on R (R = molar ratio of IL and S/Co_{mix}) at 25 °C.

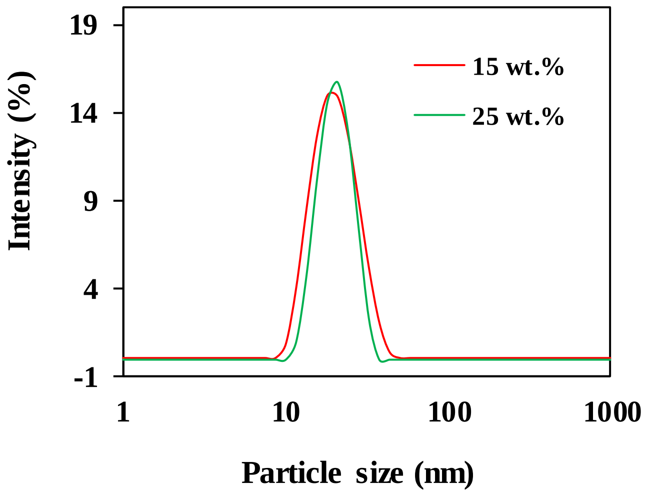

Figure S6. The size and size distribution of IL/S/Co_{mix}/IPM ME with different S/Co_{mix} concentrations (wt.%) at a 2:1 weight ratio and R = 0.2 at 25 °C.

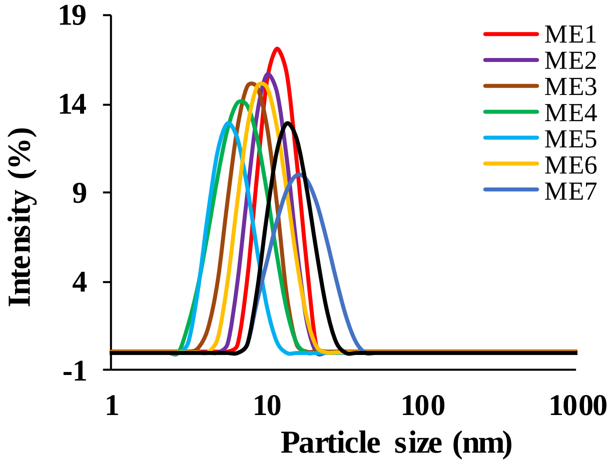

Figure S7. The size and size distribution of ACV loaded (2 mg/mL) MEs with varying S/Co weight ratios at 25 °C.

The encapsulation efficiency was calculated by the following equation:

Encapsulation efficiency (%)= (Drug concentration in ME (mg/mL) after two months)/(Drug concentration in ME (mg/mL) at initial) × 100

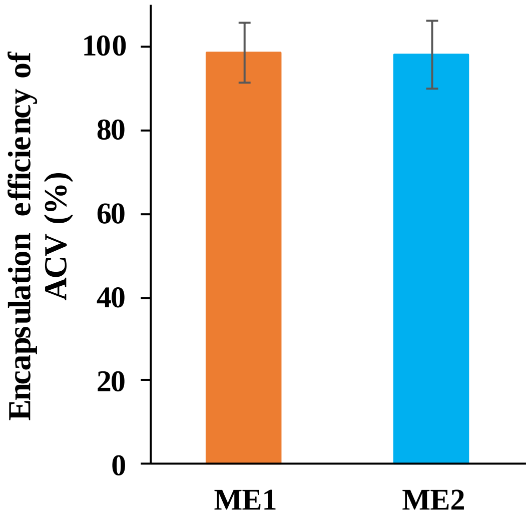

Figure S8. ACV encapsulation efficiency of MEs after two months.

Table 1S. The density and viscosity of MEs

Formulations	[a]ρ(g/cm^3)	[a]η (m Pa.s)
ME1	0.8776	23.95*,**
ME2	0.8782	22.35
ME3	0.8789	20.05
ME4	0.8797	17.1
ME5	0.8809	11.12
ME6	0.8823	21.12
ME7	0.8788	22.34
ME8	0.8795	24.74

[a]Drug-free MEs.

*compared with ME4, $p < 0.05$; **compared with ME5, $p < 0.01$; using Dunnett's multiple comparison test.

Table S2. FTIR peak shifts of SC after treatment with different MEs (mean ± SD, n = 3).

SC components		No treat	ME1		ME6		ME9	
		Absorption	Absorption	Δ_{Shift}	Absorption	Δ_{Shift}	Absorption	Δ_{Shift}
Lipid	CH_2, Asymm (cm^{-1})	2920±0.2	2924±0.5	4	2923±0.3	3	2922.5±0.3	2.5
	CH_2, Symm (cm^{-1})	2851±0.2	2854.5±0.3	3.5	2853.5±0.5	2.5	2853±0.5	2
Keratin	NH-C=O (cm^{-1})	1644±0.3	1647±0.2	3	1646.5±0.2	2.5	1646±0.3	2
		1538±0.3	1540.5±0.3	2.5	1540.3±0.4	2.3	1640±0.5	2
